# Decline of semen quality and increase of leukocytes with cigarette smoking in infertile men

**Published:** 2013-07

**Authors:** Zhi Hong Zhang, Hai Bo Zhu, Lei Lei Li, Yang Yu, Hong Guo Zhang, Rui Zhi Liu

**Affiliations:** 1 *Reproductive Medical Center, First Hospital of Jilin University, Changchun, Jilin, China.*; 2 *Department of Cell Biology, Bethune Medical College of Jilin University, Changchun, Jilin, China.*

**Keywords:** *Smoking*, *Male infertility*, *Semen analysis*, *Leukocyte*

## Abstract

**Background: **Previous researches about the effect of smoking on semen quality are contradictory, and the mechanism behind the harmful effect of smoking on semen quality still remains unclear until today.

**Objective:** The objectives of this study are evaluation of the relationship between smoking and fertility, investigation of the effects of cigarette smoking on sperm parameters and detection of presence of leukocytes within the semen of idiopathic infertile men from Northeastern China.

**Materials and Methods:** A retrospective study of 1512 infertile patients who visited affiliated hospitals of Jilin University from 2007-2010 were enrolled in this study. Patients were assigned into one non-smoking and one smoking group which was divided into mild, moderate and heavy subgroups. Sperm parameters (including leukocytes) and sperm morphology analysis were performed using standard techniques.

**Results:** Compared with non-smokers, smokers had a significant decrease in semen volumes (p=0.006), rapid progressive motility (p=0.002) and sperm viability (p=0.019); moreover, smokers had a significant increase in the levels of immotile sperms (p=0.005) and semen leukocytes (p=0.002); pH and sperm concentration were not statistically significant (p=0.789 and p=0.297 respectively). Sperm motion parameters were all lower in the smokers except for beat-cross frequency (Hz) (BCF). Further, the percentage of normal morphology sperm was decreased significantly in smokers (p=0.003), the sperm morphology was worse with increasing degree of smoking.

**Conclusion:** These findings suggest that smoking leads to a significant decline in semen quality and higher levels of leukocytes, thus smoking may affects the fertilization efficiency.

## Introduction

Drop in human semen quality has been associated with environmental factors, occupational hazards, personal habits such as drinking, diet, and smoking, and the introduction of new pathogenic factors in the population (1, 2). While some of these claims are still debated, cigarette smoking is generally accepted as a health hazard. Tobacco combustion yields about 4,000 chemical compounds. Among them, it was shown that polycyclic aromatic hydrocarbons activate a pro-apoptotic protein in mice (3). The same condition in human was shown to have deleterious effects on female embryos and male germ cells (4). 

Previous studies described the effects of smoking on semen parameters using classical microscopic analysis or computer-aided sperm analysis (CASA), with some inconsistent results. As a result, some studies described a negative effect of smoking on sperm concentration, motility, vitality, morphology and semen volume (5-11). On the other hand, other studies are not agreed with that conclusion (12-15). In 2008 Giulia proposed that insufficient number of sperm specimen could be the reason for the different results between laboratories (9). 

These inconsistencies however cause many debates. Concerning the effects of smoking on semen quality of Chinese population, only two previous studies had reported that effects, in Chongqing and Shandong province, they analyzed small sample of infertile men and large sample of fertile men respectively. In this study we retrospectively analyzed the semen quality of a large number of idiopathic infertile men, trying to evaluate the possible relationship between smoking and fertility, explore the influence of cigarette smoking (including different smoking levels) on sperm parameters and detect the presence of leukocytes within the semen of infertile patients from Northeast China.

## Materials and methods

**Participants**

A retrospective study during May 2007 to November 2010 was performed; male patients who visited the First, Second and Third Bethune Hospital of Jilin University for infertility during this period had enrolled in this study. Our daily outpatients of male infertility were about 50 patients, but most of them do not meet our inclusion criteria (Specific criteria were in the next paragraph). 

The azoospermia patients were first excluded from the study, of 1639 idiopathic infertility patients with semen analysis who were initially included in the study, 127 infertile men with factors that may affect fertility were excluded. These factors include excessive alcohol intake, chronic or hallucinatory drug use, serious systemic disease, abnormality of the external genitalia, known hereditary/familial disorders, and also excluded men involved those who had infection or trauma of the genitals. 

Enrolled participants were assigned into either a non-smoking group (n=775) or a smoking group (n=737). The smoking group was further subdivided into three subgroups: mild [smoking frequency (SF) less than 5 cigarettes per day and smoking age (SA) less than 5 years, n=180], moderate (SF 5-20 cigarettes per day and SA 5-10 years, n=327) and heavy (SF >20 cigarettes per day and SA >10 years, n=230), per cigarette contains about 1g tobacco. Values of normal sperm parameters advised by World Health Organization (WHO, 1999) guidelines were applied as a control for semen analysis and determination of abnormal fertility (16). This study was approved by the Reproductive Medicine Ethics Committee of First Hospital of Jilin University, and all patients signed informed consents of this study before semen analysis.

**Sample collection and routine semen analysis**

Participants in this study were asked to collect their semen samples by masturbation at the hospital in a polypropylene container after 2-7 days of sexual abstinence. The semen were then allowed to liquefy at 37^o^C and processed immediately thereafter using the WHO recommended guidelines (WHO, 1999). 

Briefly, the semen volume and pH measurement were recorded, sperm viability was detected using eosin staining method for the determination of live sperms. Sperm motility parameters of the semen specimens were obtained after preheating at 37^o^C using a quality detector counting board and a semen color automatic analysis system (WLJY-9000, Beijing, China). Leukocyte count was evaluated under light microscopy using benzidine staining and adjusted according to the recorded volume of ejaculated semen (count/ml of peroxidase positive leukocyte). 

**Sperm morphology analysis**

Blind evaluation of sperm morphology by two in house trained technicians was obtained from air-dried smears fixed and stained with Wright-Giemsa (Diff-Quick; Risheng Reproductive Medicine Science Tech. Co. Ltd, ChangChun, China) using WHO 1999 Tygerberg’s strict criteria.

**Statistical analysis**

Statistical analysis was carried out using software package SPSS 11.5 (SPSS Inc, Chicago, IL). First, homogeneity of variance test was used to variables between the pooled, mild, moderate and heavy smokers and the non-smokers. If variance was homogeneity, two independent samples T-test was used; if variance was not homogeneity, two independent sample rank sum test was used. All data were presented as Mean±SD, sperm morphology shown in table 3 were relative percentages. P<0.05 was considered statistically significant. 

## Results

Among 1639 patients, a total of 1512 infertile men from Northeastern China met the inclusion criteria and were recruited to evaluate the impact of smoking on sperm infertile condition. They include 775 non-smokers and 737 smokers (180 mild smokers, 327 moderate smokers, and 230 heavy smokers). The age of smokers and non-smokers had no statistically significant difference (p=0.872), (Table I). Semen parameters (semen volume, pH, sperm concentration, motility, viability, leukocytes and sperm motion parameters) in smokers and non-smokers were compared in all 1512 participants (Table II, and III). Since not every patient has sperm morphology data, so we only studied 615 non-smokers and 562 smokers (127 mild smokers, 242 moderate smokers, and 139 heavy smokers) with sperm morphology analysis, (Table IV). 

As shown in table II, semen volumes of smokers were significantly lower than those of non-smokers (p=0.006). The fast progressive motility of their sperm (grade a and grade a+b) and viability rate were also significantly lower (p=0.002, p=0.004, and p=0.019 respectively). On the other hand, immotile sperms (grade d) and presence of leukocytes in semen were significantly increased among smokers (p=0.005, p=0.002 respectively). Compare with non-smokers, pH and sperm concentration were lower in smokers, but this differences were not statistically significant (p=0.789 and p=0.297 respectively). In subgroups, compared with non-smokers, seminal leukocytes gradually increased with increasing degree of smoking, the p-value of the mild, moderate and heavy subgroups were p=0.241, p=0.025 and p=0.001 respectively.

For motion parameters, the study results showed that all parameters were lower in the smokers except for beat-cross frequency (Hz) (BCF), in other words, the effect of smoking on BCF was not noticeable and not significant. While average path velocity (VAP), linearity (LIN), wobble (WOB) and straightness (STR) were statistically significant (p=0.020, p=0.024, p=0.006 and p=0.035 respectively). In the subgroups, levels of curvilinear velocity (VCL), straight-line velocity (VSL), VAP, amplitude of lateral head displacement (ALH), LIN and STR showed a decreasing trend associated with the increased level of smoking. In addition in heavy smoker subgroup, VSL and VAP showed significant decrease compared with non-smokers (p=0.006 and p=0.006 respectively) as shown in table III. 

The percentage of normal sperm morphology was significantly lower in smokers compared with non-smokers (p=0.003). similarly the percentage of neck defects decreased; head and tail defects increased, which the difference in head defects were statistically significant (p=0.016). Compare with non-smokers the percentage of large head, pear-shaped head, round head, amorphous head, acrosomal area small and double head showed increase, in which, only difference in acrosomal area small was statistically significant (p=0.041); small head and tapered head showed decrease. In subgroups, compared with non-smokers, normal sperm morphology rates decreased with increasing degree of smoking, the p-value of the mild, moderate and heavy subgroups were p=0.437, p=0.022 and p=0.001 respectively as shown in Table IV.

**Table I:** Age and groups of the enrolled participants (Mean ± SD)

	**Excluded**	**Non-smokers**	**Smokers**			
			**Total**	**Mild**	**Moderate**	**Heavy**
Number	127	775	737	180	327	230
Age (years)	30.23 ± 6.23	29.91 ± 4.81	29.60 ± 4.84	29.81 ± 4.89	29.74 ± 5.17	30.02 ± 4.75
p-value	**-**	**-**	0.872	0.064	0.640	0.589

Two independent samples T-test was used. P-values result was compared with non-smokers

**Table II:** Sperm parameters in non-smokers and smokers groups (Mean ± SD)

**Sperm parameters**		**Non-smokers** **(n=775** **)**	**Smokers**				
			**Total** ** (** **n=737** **)**	**p-value**	**Mild** ** (** **n=180** **)**	**Moderate (n=327** **)**	**Heavy (n=230** **)**
Volume (ml)		3.46 ± 1.66	3.21 ± 1.49 **	0.006	3.16 ± 1.55*	3.20 ± 1.44*	3.26 ± 1.52
pH		7.13 ± 0.23	7.12 ± 0.24	0.789	7.13 ± 0.24	7.12 ± 0.23	7.11 ± 0.23
Concentration (×10^6^/ml)		57.02 ± 46.72	54.43 ± 50.7	0.297	55.10 ± 49.35	54.64 ± 51.34	53.56 ± 49.13
Viability (%)		55.25 ± 18.79	52.91 ± 20.21*	0.019	53.49 ± 19.43	53.31 ± 20.48	51.84 ± 20.55*
Leukocytes (×10^6^/ml)		0.097 ± 0.016	0.120 ± 0.188**	0.002	0.101 ± 0.158	0.124 ± 0.198*	0.132 ± 0.199**
Motility grade							
	a (%)	16.38 ± 11.84	14.71 ± 11.76**	0.002	15.54 ± 11.67	14.51 ± 11.81*	14.23 ± 11.87*
	b (%)	8.23 ± 5.20	7.57 ± 5.47*	0.017	7.26 ± 5.44*	7.86 ± 5.70	7.47 ± 5.18
	c (%)	6.48 ± 5.42	6.12 ± 5.83	0.212	5.84 ± 5.17	6.54 ± 6.52	5.80± 5.37
	d (%)	68.92 ± 19.60	71.66 ± 19.80**	0.005	71.36 ± 18.65	71.22 ± 20.85	72.53 ± 19.37*
	(a + b) (%)	24.61 ± 15.68	22.28 ± 15.65**	0.004	22.80 ± 15.03	22.37 ± 16.17*	21.70 ± 15.52*

Two independent samples T-test or rank sum test was used. P-values result was compared smokers with non-smokers.

* p<0.05,

** p<0.01.

**Table III:** Sperm motion parameters in non-smokers and smokers groups (Mean ± SD

**Motion parameters**	**Non-smokers** **(** **n=775** **)**	**Smokers**				
		**Total** ** (** **n=737** **)**	**p-value**	**Mild** ** (** **n=180** **)**	**Moderate (n=327** **)**	**Heavy (n=230** **)**
VCL (μm/s)	44.03 ± 9.22	41.89 ± 13.25	0.100	43.31 ± 13.68	41.78 ± 11.97*	40.78 ± 14.40*
VSL (μm/s)	28.86 ± 6.70	27.28 ± 10.05	0.071	28.32 ± 10.39	27.33 ± 9.29	26.27 ± 10.66**
VAP (μm/s)	31.75 ± 6.84	29.80 ± 10.30*	0.020	30.83 ± 10.77	29.71 ± 9.36*	29.00 ± 11.04**
MAD (°)	53.75 ± 9.29	51.24 ± 14.93	0.063	51.07 ± 15.07	51.39 ± 13.91	51.20 ± 15.49
ALH (μm)	3.04 ± 2.76	2.80 ± 1.88	0.050	2.87 ± 1.72	2.79 ± 2.22	2.75 ± 1.46
BCF (Hz)	5.09 ± 1.10	5.10 ± 1.93	0.717	5.00 ± 2.03	5.18 ± 1.77	5.07 ± 2.04
LIN (%)	64.12 ± 9.61	60.24 ± 17.11*	0.024	61.11 ± 17.49	60.75 ± 15.64*	58.76 ± 18.61*
WOB (%)	71.75 ± 7.86	67.16 ± 18.31**	0.006	67.58 ± 18.59	67.65 ± 17.44*	66.11 ± 19.22*
STR (%)	86.85 ± 7.58	81.59 ± 21.60*	0.035	82.80 ± 21.77	81.38 ± 20.91*	80.78 ± 22.38*

Two independent samples T-test or rank sum test was used. P-values result was compared smokers with non-smokers.

* p<0.05,

** p<0.01.

**Table IV:** Sperm morphology in non-smokers and smokers groups (%, Mean ± SD)

**Sperm morphology**	**Non-smokers** **(** **n=615** **)**	**Smokers**				
		**Total** ** (** **n=562** **)**	**p-value**	**Mild** ** (** **n=127** **)**	**Moderate** ** (** **n=242** **)**	**Heavy** ** (** **n=139** **)**
Normal form	11.17 ± 3.56	10.61 ± 3.69**	0.003	10.95 ± 3.41	10.61 ± 3.80*	10.31 ± 3.76**
Head defects	87.81 ± 3.78	88.32 ± 4.30*	0.016	87.70 ± 4.54	88.40 ± 3.86*	88.75 ± 4.60**
Large head	7.28 ± 7.50	7.64 ± 7.60	0.502	7.55 ± 6.68	7.30 ± 7.50	8.18 ± 8.47
Small head	7.64 ± 6.43	7.34 ± 6.84	0.147	7.23 ± 5.24	7.41 ± 7.08	7.36 ± 7.73
Tapered head	15.60 ± 12.03	14.61 ± 12.37	0.064	15.62 ± 14.76	14.35 ± 11.69	14.06 ± 10.84
Pear-shaped head	13.19 ± 12.03	13.22 ± 10.10	0.842	12.70 ± 9.37	14.14 ± 10.51	12.45 ± 10.11
Round head	4.38 ± 4.49	4.48 ± 4.19	0.584	4.29 ± 5.26	4.21 ± 3.24	4.96 ± 4.23*
Amorphous head	39.57 ± 13.39	40.77 ± 15.01	0.329	40.12 ± 16.93	40.77 ± 15.01	41.39 ± 13.17
Vacuolated head	0.03 ± 0.17	0.03 ± 0.21	0.488	0.04 ± 0.19	0.02 ± 0.12	0.06 ± 0.30
Acrosomal area small	0.08 ± 0.43	0.16 ± 0.64*	0.041	0.09 ± 0.45	0.14 ± 0.54*	0.25 ± 0.86*
Double heads	0.05 ± 0.21	0.06 ± 0.24	0.134	0.06 ± 0.24	0.06 ± 0.22	0.07 ± 0.25
Neck defects	2.34 ± 2.29	2.15 ± 2.02	0.222	1.94 ± 1.90	2.34 ± 2.12	2.08 ± 1.97
Tail defects	2.24 ± 1.71	2.30 ± 2.21	0.114	2.43 ± 2.49	2.27 ± 2.15	2.24 ± 2.01

Two independent samples T-test or rank sum test was used. P-values result was compared smokers with non-smokers. A sperm may simultaneously have head, neck and tail defects.

* p<0.05,

** p<0.01.

## Discussion

Many previous studies have examined the influence of cigarette smoking on semen quality in infertile men, but many of the results are still debated. Here, we studied 775 non-smokers and 737 smokers (180 mild smokers, 327 moderate smokers, and 230 heavy smokers) with idiopathic infertility. In general, the age of smokers and non-smokers had no statistically significant (p=0.872), and alcohol consumption was not considered in the analysis as it was moderate and has been shown to have no negative effect on fertility in men or women (17, 18). 

Although several studies have reported that the mutagenic components of cigarette smoke adversely affect rapidly dividing cells, including germ cells (19). Comparison of variables between infertile smokers and non-smokers revealed significant parameters differences with motility, semen volume, viability and sperm morphology. Our results were consistent with previous literature reports (8, 20). However this study also describes a smoking dosage element showing higher percentage of rapidly progressive sperm defect in heavy smokers when compared to non-smokers. 

Except for sperm concentration, other sperm parameters in men with idiopathic infertility were not affected by smoking which support the results observed by Collodel (21). We showed that although the degree of smoking had an increasingly negative impact on sperm quality, not all sperm parameters were affected. It is possible that the absence of statistically significant between smokers and non-smokers was, in some cases, hidden in the background produced as a result of long term passive smoking among the non-smokers (non-smoker exposure to second-hand smoke). Sperm concentration and pH can be excluded from this proposal as these results did not deviate from the normal range (WHO, 1999 edition) (16). 

Actually most results showed that smoking mostly affected semen quality such as sperm progressive motility and viability. In the smoking groups, most sperm motion parameters (VCL, VSL, VAP, ALH, LIN and STR) showed significant decreasing trend with increasing level of smoking. On the other hand, BCF did not show variation across the groups. Sperm heads are the reference indicators for VCL, VSL and VAP which indicate movement speed of head, actual and relative displacement to describe the ability of sperm motility. When there is tail defect, the sperms swing ability is also reduced; here sperm progressive movement and sperm tail side movement ability were both decreased, and most motion parameters (VCL, VSL, VAP, LIN, WOB and STR) and highest decline of motility was seen among the heavy smokers. 

In this study, decline of progressive motility, movement velocity and lower sperm viability were mostly seen among smoking men. These typical signs of reduced semen quality are critical at the time of fertilization as they are assist in favoring the passage of the sperm through the pellucid zone, in other words, without this assistance a non-motile or abnormally-motile sperm would not be able to fertilize the egg.

The percentage of normal morphology sperm was significantly lower in smokers compare with non-smokers (p=0.003), neck defects showed decrease, head and tail defects showed increase, and head defects changes was statistically significant (p=0.016). These observations suggest that smoking may mainly affects sperm head morphology and support previous reports by Kunzle and Elshal, especially in men who are heavy smokers and/or who have smoked for many years (5, 22, 23). 

None the less, these observations are debated by others (12, 20). Further researches have shown that many compounds in cigarette smoke in addition to nicotine may affect semen parameters and sperm morphology and that the degree of sperm abnormality and smoking are correlated (22, 24). These findings are supported here as we showed that the degree of smoking was directly associated to an overall change of sperm parameters including their morphology, and worsened successful fertilization (25). 

Our study also showed that seminal leukocytes concentration was higher in smoking groups (p=0.002), with increasing degree of smoking, seminal leukocytes gradually increased [p=0.241 (mild), p=0.025 (moderate) and p=0.001 (heavy) respectively]. Actually, it was shown that nicotine concentrations of 400-800 ng/ml has a detrimental effect on sperm motility, membrane function, and the ability to undergo the physical changes necessary to fertilize the egg (26). However, the mechanism explaining how leukocytes interact with sperm is not yet clear. 

Alternatively, another adverse effect on sperm quality could be the amount of reactive oxygen species (ROS) effect on sperm quality, since elevated levels of ROS have been found in infertile smoking men, and concomitantly, decreasing levels of antioxidants in seminal plasma (25, 27, 28). Furthermore, in 2003, Horak reported that oxidants in cigarette smoke are thought to damage sperm DNA, and that smokers have more oxidative DNA damage in their sperm than non-smokers (29). Here we propose that, smoking may increase the concentration of leukocytes in semen, and hence in correlation with sperm motility may damage semen quality. 

Perhaps stopping smoking and increasing antioxidant (reduce DNA damage) intake should be recommended to all infertile smokers. In summary, smoking adversely affects semen quality, with adverse consequences on sperm morphology and decline sperm motility. Increased leukocytes, but the role of leukocytes in semen remains undetermined. In short, the decline in semen quality may be related to smoking, and lead to male infertility. 
